# JWST detection of a supernova associated with GRB 221009A without an r-process signature

**DOI:** 10.1038/s41550-024-02237-4

**Published:** 2024-04-12

**Authors:** Peter K. Blanchard, V. Ashley Villar, Ryan Chornock, Tanmoy Laskar, Yijia Li, Joel Leja, Justin Pierel, Edo Berger, Raffaella Margutti, Kate D. Alexander, Jennifer Barnes, Yvette Cendes, Tarraneh Eftekhari, Daniel Kasen, Natalie LeBaron, Brian D. Metzger, James Muzerolle Page, Armin Rest, Huei Sears, Daniel M. Siegel, S. Karthik Yadavalli

**Affiliations:** 1 Center for Interdisciplinary Exploration and Research in Astrophysics (CIERA), Northwestern University, Evanston, IL USA; 2 Center for Astrophysics | Harvard & Smithsonian, Cambridge, MA USA; 3 Department of Astronomy, University of California, Berkeley, CA USA; 4 Department of Physics & Astronomy, University of Utah, Salt Lake City, UT USA; 5 Department of Astrophysics/IMAPP, Radboud University, Nijmegen, The Netherlands; 6 Department of Astronomy & Astrophysics, The Pennsylvania State University, University Park, PA USA; 7 Institute for Gravitation and the Cosmos, The Pennsylvania State University, University Park, PA USA; 8 Institute for Computational & Data Sciences, The Pennsylvania State University, University Park, PA USA; 9 Space Telescope Science Institute, Baltimore, MD USA; 10 Department of Physics, University of California, Berkeley, CA USA; 11 Department of Astronomy/Steward Observatory, Tucson, AZ USA; 12 Kavli Institute for Theoretical Physics, University of California, Santa Barbara, CA USA; 13 Department of Physics and Columbia Astrophysics Laboratory, Columbia University, New York, NY USA; 14 Center for Computational Astrophysics, Flatiron Institute, New York, NY USA; 15 Department of Physics and Astronomy, Northwestern University, Evanston, IL USA; 16 Institute of Physics, University of Greifswald, Greifswald, Germany; 17 Department of Physics, University of Guelph, Guelph, Ontario Canada

**Keywords:** Transient astrophysical phenomena, Time-domain astronomy, High-energy astrophysics

## Abstract

Identifying the sites of r-process nucleosynthesis, a primary mechanism of heavy element production, is a key goal of astrophysics. The discovery of the brightest gamma-ray burst (GRB) to date, GRB 221009A, presented an opportunity to spectroscopically test the idea that r-process elements are produced following the collapse of rapidly rotating massive stars. Here we present James Webb Space Telescope observations of GRB 221009A obtained +168 and +170 rest-frame days after the gamma-ray trigger, and demonstrate that they are well described by a SN 1998bw-like supernova (SN) and power-law afterglow, with no evidence for a component from r-process emission. The SN, with a nickel mass of approximately 0.09 *M*_⊙_, is only slightly fainter than the brightness of SN 1998bw at this phase, which indicates that the SN is not an unusual GRB-SN. This demonstrates that the GRB and SN mechanisms are decoupled and that highly energetic GRBs are not likely to produce significant quantities of r-process material, which leaves open the question of whether explosions of massive stars are key sources of r-process elements. Moreover, the host galaxy of GRB 221009A has a very low metallicity of approximately 0.12 *Z*_⊙_ and strong H_2_ emission at the explosion site, which is consistent with recent star formation, hinting that environmental factors are responsible for its extreme energetics.

## Abstract (web summary)

JWST observations of GRB 221009A reveal the associated supernova, confirming that the GRB resulted from the collapse of a rapidly rotating massive star. The lack of r-process emission suggests that these extreme events are not key sources of the heaviest elements.

## Main

The origin of the heaviest elements in the Universe, specifically those formed by means of rapid neutron capture (r-process) nucleosynthesis, remains a major open question in astrophysics^1,2^. Given the high density of neutron-rich material needed for the r-process to occur, the collisions of neutron stars have long been a suspected source^3,4^ and, indeed, the observations of the kilonova associated with GW 170817 confirmed that binary neutron star (BNS) mergers are the source of at least some of the r-process material in the Universe^5–9^. However, there is growing evidence that there may be multiple sites of r-process nucleosynthesis from studies of low-metallicity galactic halo stars, dwarf galaxy and globular cluster enrichment^10–14^.

A second proposed site of the r-process is in rapidly rotating cores of massive stars that collapse into an accreting black hole, producing similar conditions as the aftermath of a BNS merger^15^. Theoretical simulations suggest that accretion disk outflows in these so-called ‘collapsars’ may reach the neutron-rich state required for the r-process to occur^15,16^. The larger mass of r-process material synthesized per event compared with BNS mergers suggests that collapsars could be a dominant source, making them a possible missing piece in our understanding of r-process enrichment in the Universe.

The discovery of the long-duration gamma-ray burst (GRB) GRB 221009A, the brightest GRB ever observed^17–19^, on 9 October 2022 at a relatively nearby redshift of *z* = 0.151 (ref. ^20^) presents a unique opportunity to search for r-process signatures in a collapsar. Collapsars are the favoured explanation for long GRBs (LGRBs), which result from the launch of a relativistic jet and its subsequent interaction with the surrounding medium^21–23^. r-Process nucleosynthesis is more likely to occur in collapsars with large accretion disk masses, which are also thought to be linked with brighter GRBs^15^, making GRB 221009A a particularly strong candidate to search for r-process signatures. These events are known to be accompanied by broad-lined type Ic supernovae (SNe Ic-BL) characterized by higher velocities than normal type Ic supernovae, suggesting that the energy powering LGRBs also affects the associated supernovae (see ref. ^24^ for a review).

It is the supernova (SN) following a LGRB that would be responsible for carrying r-process material from the explosion site into the interstellar medium. Although early-time observations of GRB 221009A provided an exquisite view of the afterglow^25–27^, to date, there are conflicting claims in the literature regarding the presence of an associated SN, which are due, in part, to the bright afterglow and high Milky Way extinction^28–31^. Moreover, there have been claims that two recent LGRBs are associated with BNS mergers^32–35^, making the search for an SN associated with GRB 221009A crucial not only for an r-process search, but also for understanding the origin of its extreme luminosity.

Here, we present late-time James Webb Space Telescope (JWST) observations of GRB 221009A consisting of a near-infrared (NIR) spectrum and imaging in four NIR bands. These observations provide clear detection of an SN associated with this extreme event and enable the search for r-process emission in a nebular-phase spectrum of a GRB-SN. Moreover, these data provide a detailed NIR view of the host galaxy, enabling an assessment of environmental factors that may be responsible for this extraordinary GRB.

## Identification of SN emission

**Fig. 1:**
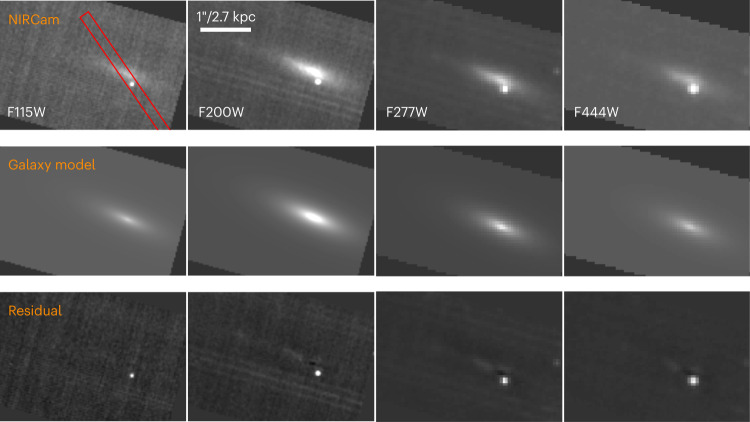
JWST/NIRCam imaging. Our images of GRB 221009A (top row), best-fit GALFIT galaxy models (middle row) and GALFIT model subtracted images (bottom row). Images are shown with north up and east to the left. A clear point source is detected at the location of GRB 221009A. The red rectangle shows the NIRSpec slit orientation. PSF photometry of GRB 221009A was performed on the galaxy-subtracted images. The host galaxy is well described by a single Sérsic component, although some residual galaxy structure remains in the F200W, F277W and F444W filters.

**Extended Data Fig. 1:**
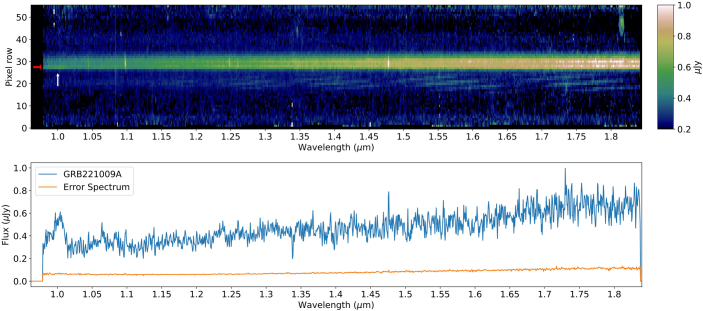
NIRSpec/G140M Spectrum. Top: Final combined 2D NIRSpec/G140M spectrum of GRB 221009A resulting from our re-reduction. The trace is clearly dominated by the spatially resolved host galaxy. A broad emission feature is visible near ≈ 1*μ*m (white arrow) at the expected spatial location of GRB 221009A (red arrow). The background below the trace contains structure that is likely due to the diffraction spike of a nearby star. Spatially resolved emission lines from the host galaxy are also detected. Bottom: 1D spectrum of GRB 221009A extracted via a two-component Gaussian fit to the spatially resolved trace to isolate the spectrum of GRB 221009A from the host galaxy contribution.

**Extended Data Fig. 2:**
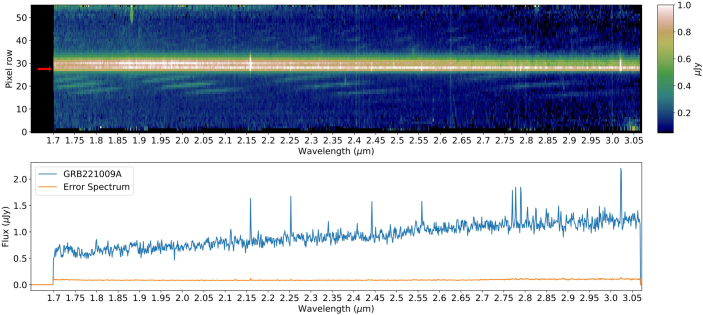
NIRSpec/G235M Spectrum. Top: Final combined 2D NIRSpec/G235M spectrum of GRB 221009A resulting from our re-reduction. The trace from GRB 221009A (red arrow) is more clearly visible at the red end. Bottom: 1D spectrum of GRB 221009A extracted via a two-component Gaussian fit to the spatially resolved trace to isolate the spectrum of GRB 221009A from the host galaxy contribution. Notably, several host galaxy emission lines appear to be stronger at the position of GRB 221009A than the rest of the galaxy, resulting in significant excess flux from these lines appearing in the GRB 221009A spectrum (see Fig. 5 for line identifications).

**Extended Data Fig. 3:**
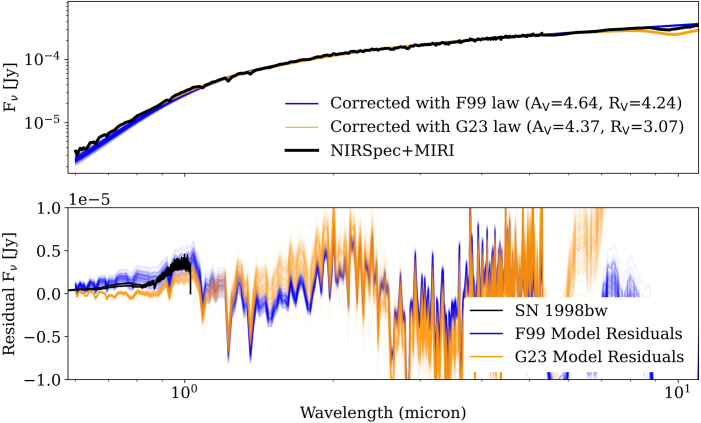
Extinction Fits to the Early JWST Data. Top: Comparison of the early-time NIRSpec/PRISM and MIRI spectra (black) along with two models for the afterglow and dust law ([37] in blue, and [38] in orange). Each line of the model represents a draw from the posterior. Note that there is a silicate feature at ≃ 10*μ*m which is not properly modeled without a detailed dust composition; here, we fit *λ* < 8*μ*m to avoid this feature. Bottom: Model residuals compared to SN 1998bw (black; [40]). Again, individual lines represent independent draws from the model posterior. For both laws, strong systematic residuals are found across the full wavelength range. An SN 1998bw-like supernova cannot be ruled out.

We obtained spectroscopy with the Near Infrared Spectrograph (NIRSpec) using the medium-resolution gratings covering 1–3 μm on 20 April 2023 and imaging with the Near Infrared Camera (NIRCam) using the F115W, F200W, F277W and F444W filters on 22 April 2023. These observations occurred +194 and +196 observer-frame days after the burst (rest-frame phases of +168 and +170 days, respectively). The afterglow of GRB 221009A is clearly detected in our images, from which we measured photometry (Fig. 1; see Methods for details). In our NIRSpec observations, we detect a clear spectral trace containing flux from GRB 221009A and its host galaxy (Extended Data Figs. 1 and 2; see Methods for details of the spectral extraction). Owing to the high Milky Way extinction^36^ and possible non-negligible extinction intrinsic to the host galaxy^19,20,31^, we analysed archival early-phase NIRSpec/PRISM and Mid-Infrared Instrument (MIRI) spectra^31^ of GRB 221009A using multiple dust laws to constrain the extinction (Methods and Extended Data Fig. 3). We used the resulting extinction parameters (Extended Data Table 1) to correct our rest frame +168 day NIRSpec grating spectrum.

**Fig. 2:**
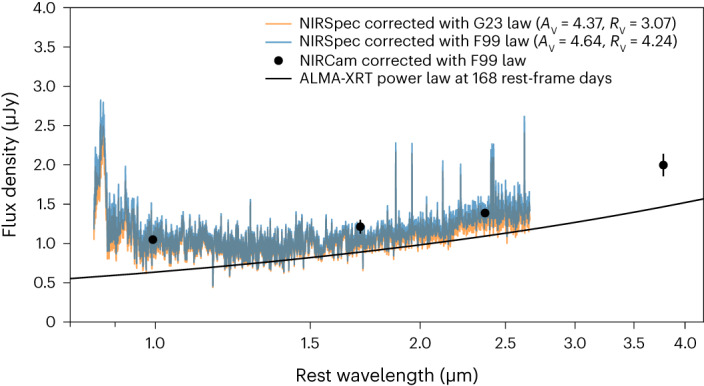
JWST/NIRSpec spectrum. Our +168 rest-frame phase JWST/NIRSpec G140M+G235M spectrum of GRB 221009A corrected for extinction (see Methods for spectral extraction details; error spectra are shown in Extended Data Figs. 1 and 2). We show two versions corrected using the G23 (orange)^38^ and F99 (blue)^37^ extinction laws and corresponding best-fit extinction parameters from fitting the early-time NIRSpec/PRISM and MIRI data from ref. ^31^ as described in Methods ‘Constraints on foreground dust from early-time spectroscopy’. In both cases, the spectrum appears to exhibit multiple components, with SN-like emission at *λ* ≲ 1.5 μm and rising flux at *λ* ≳ 1.5 μm, which is likely to be due to the GRB afterglow power law. We also show our JWST/NIRCam photometry corrected using the F99 extinction law^37^ (points with 1*σ* error bars), as well as the ALMA-XRT power law (black line).

In Fig. 2, we show two versions of the spectrum, one corrected using an extinction curve from ref. ^37^ and another using one from ref. ^38^, transformed to the rest frame of GRB 221009A. In both cases, the spectrum exhibits an overall flat shape in the range ~1–1.5 μm, with a smooth, gradual upturn at redder wavelengths extending to the edge of our coverage at ~2.7 μm and a sharp upturn at bluer wavelengths due, in part, to apparent broad emission features. The use of different extinction laws and parameters, within the range of uncertainties from our fitting, does not change these fundamental characteristics.

The gradual rise in the spectrum at wavelengths *λ* ≳ 1.5 μm strongly resembles a power-law shape and therefore this region is likely to contain a significant contribution from the afterglow of GRB 221009A. In addition, our photometric observation in the F444W filter (which lies redward of our spectroscopic coverage) indicates that the flux continues to rise at longer wavelengths (≳3.8 μm, rest frame). The fluxes measured in the F200W, F277W and F444W filters are consistent with a single power law with an index of *β* = −0.64 ± 0.10. The shape of the NIRSpec spectrum at wavelengths *λ* ≳ 1.5 μm is slightly steeper than this slope, with a power-law index of *β* = −0.76 ± 0.07, although it is consistent within the uncertainties.

**Extended Data Fig. 4:**
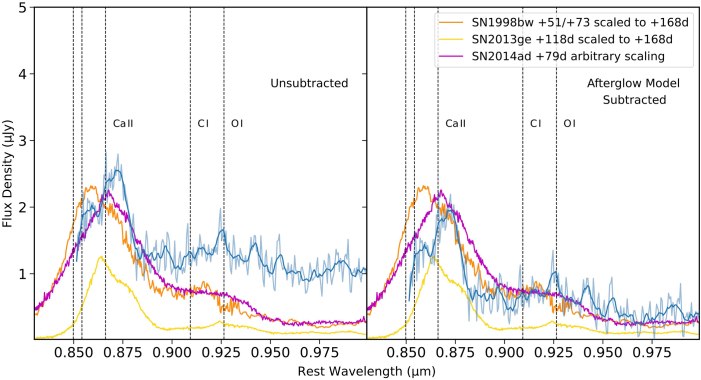
Broad SN Features. Left: Zoom-in on the blue end of our spectrum of GRB 221009A highlighting the broad emission features we attribute to the CaII NIR triplet and OI. Also shown are comparison spectra of SN 1998bw (orange) and SN 2013ge (gold) both scaled to the distance of GRB 221009A and their brightness at the phase of our observations, as well as SN 2014ad (magenta) arbitrarily scaled. Right: Spectrum of GRB 221009A after subtracting our best-fit afterglow model from the joint SN+afterglow fit described by the power law *F*_*ν*_ ∝ *ν*^−0.76±0.07^.

At *λ* ≲ 1.5 μm, the spectrum clearly deviates from an extrapolation of the power law at *λ* ≳ 1.5 μm, exhibiting an overall flat shape and several broad SN-like emission features. Indeed, we identify two broad emission features located at wavelengths of ~0.86 μm and ~0.92 μm, which are consistent with the Ca ii NIR triplet and O i, respectively. These are typical nebular-phase emission lines observed in core-collapse supernovae (for example, ref. ^39^). We show a zoomed-in comparison of these features with those seen in SN 1998bw, SN 2013ge and SN 2014ad in Extended Data Fig. 4. In addition to the flat spectral shape at ~1–1.5 μm, these emission features strongly support the identification of SN emission in our spectrum of GRB 221009A. Our observation, therefore, represents the latest phase NIR spectrum of an SN associated with a GRB to date.

### Isolating the SN signal

**Extended Data Fig. 5:**
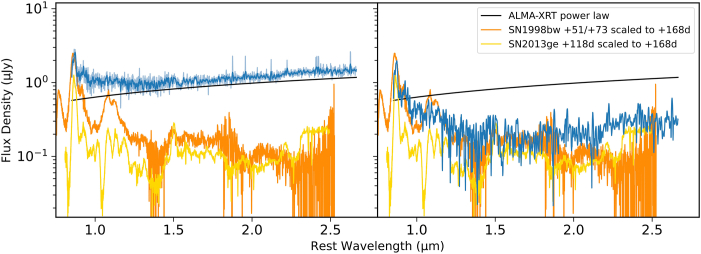
Comparisons with Previous SNe and Assessment of the ALMA-XRT Power Law. Left: Our NIRSpec spectrum of GRB 221009A, corrected for extinction using the law from ref. ^37^ and best-fit parameters from the fitting described in the Methods (blue), compared to ground-based late-time NIR spectra of SN 1998bw (orange) and SN 2013ge (gold) scaled to the distance of GRB 221009A and the same phase of our observations. Right: Comparison between SN 1998bw and SN 2013ge and our spectrum of GRB 221009A (smoothed) after subtracting an estimate of the afterglow contribution as described by the power law connecting ALMA and Swift/XRT observations taken around the same phase as our JWST data (*F*_*ν*_ ∝ *ν*^−0.63^; black line). Significant flux rising toward the red remains in the resulting subtracted spectrum, inconsistent with the comparison SNe, indicating this model likely does not adequately describe the afterglow contribution at these wavelengths.

Although disentangling the SN and afterglow components is not straightforward, the relative featureless nature of the red end of the spectrum indicates that the afterglow component is sufficiently bright to not only affect the overall shape but also to dilute SN features with respect to the continuum in that region (see Methods and Extended Data Fig. 5 for comparisons with previous supernovae).

To separate the afterglow and SN components, we considered several afterglow models. First, we used Atacama Large Millimeter/submillimeter Array (ALMA) and Swift X-ray Telescope (Swift/XRT) observations obtained at roughly the same phase as our NIRSpec spectrum and modelled the afterglow at NIR wavelengths as a power law connecting the radio and X-ray data. We find flux density *F*_*ν*_ ∝ *ν*^−0.63 ± 0.03^ for frequency ν (see Methods for details). We show this power law, normalized to the measured radio and X-ray flux, compared with our spectrum in Fig. 2. While the ALMA-XRT power-law slope is similar to the shape of our spectrum at *λ* ≳ 1.5 μm, our data is systematically offset to higher flux, which indicates that the ALMA-XRT power law does not fully capture the afterglow contribution at NIR wavelengths. Moreover, the implied SN component deviates from the expected spectral shape of an SN (Methods and Extended Data Fig. 5).

Next, we modelled the afterglow from our spectrum itself, namely, as a power law with a slope determined by fitting our spectrum at wavelengths *λ* ≳ 1.5 μm, where the afterglow is likely to be dominating. We find a best-fit power law of *F*_*ν*_ ∝ *ν*^−0.76 ± 0.07^. This is steeper than the ALMA-XRT power law, which further confirms that interpolating the millimetre and X-ray bands is not likely to provide the best representation of the afterglow at these wavelengths. We then performed a joint fit of an SN template and the fitted power law, with the power-law slope fixed, to determine the best-fit combination of SN and afterglow. For the SN template, we used the +51 day spectrum of SN 1998bw as this is the latest available NIR spectrum of another GRB-SN^40^, allowing the overall flux normalization to vary.

**Fig. 3:**
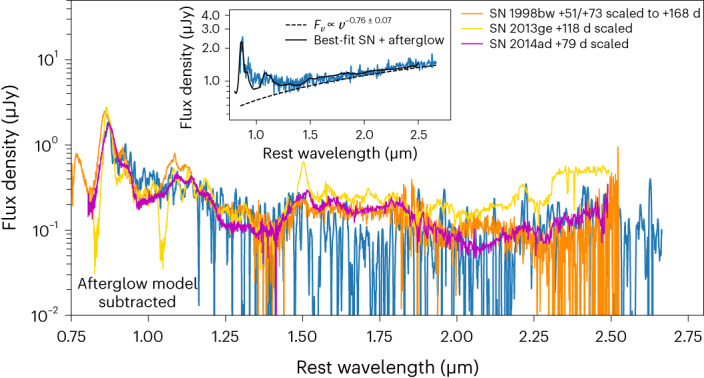
Spectral fit and comparisons. Our NIRSpec spectrum of GRB 221009A (blue, smoothed) after subtracting our best-fit afterglow model. The unsubtracted spectrum, best-fit afterglow model (dashed black line) and best-fit SN + afterglow (black line) are shown in the inset. We show late-time spectra of SN 2013ge (gold^42^) and SN 2014ad (magenta^39^) scaled to match the shape and features of the afterglow-subtracted spectrum at *λ* ≲ 1.5 μm where the SN dominates, demonstrating the overall resemblance with these comparison supernovae Ic/Ic-BL. We also show SN 1998bw (orange^40^) scaled to the distance of GRB 221009A and the phase of our spectrum, showing that it matches not only the shape but the overall flux level of our spectrum. The close match with supernovae Ic-BL, in particular, demonstrates the presence of a typical GRB-SN in our spectrum.

In Fig. 3, we present the best-fit SN 1998bw + afterglow spectrum and our spectrum of GRB 221009A after subtracting the best-fit afterglow component. We compare our afterglow-subtracted spectrum with the SN 1998bw spectrum scaled to the distance of GRB 221009A and the brightness of SN 1998bw at the phase of our JWST spectrum using the light curve of SN 1998bw from ref. ^41^. The best-fit SN component is ~30% fainter than the expected brightness SN 1998bw would have at this distance and phase. We also compare with late-time spectra of the SN Ic SN 2013ge^42^ and the SN Ic-BL SN 2014ad^39^. To directly compare the shapes and features we scaled SN 2013ge and SN 2014ad to best match the spectral shape and features at the blue end of the afterglow-subtracted spectrum where the SN component dominates.

These events provide an excellent visual match to the afterglow-subtracted spectrum, which confirms that our estimate of the afterglow contribution is reasonable. In addition, the inferred ratio of Ca ii/O i is a much better match to the ratios seen in the three comparison objects compared with the case of no afterglow subtraction (Extended Data Fig. 4). Although the width of the Ca ii emission complex exhibits a better match with SN 2013ge, the afterglow-subtracted spectrum does not show the same strong absorption seen at ~1.1 μm in SN 2013ge, possibly due to the SN associated with GRB 221009A having a higher ejecta velocity. SN Ic-BL-like velocities are further supported by the better overall match to SN 2014ad and SN 1998bw. The narrower width of Ca ii compared with SN 2014ad and SN 1998bw may be an artefact of the instrumental response affecting the shape at the blue end of the line. We also identify evidence for a broad emission feature near *λ* ≈ 1.5 μm, which is consistent with the location of the 1.503 μm line of Mg i seen in the comparison objects and large samples of other supernovae Ic/Ic-BL^39^.

In summary, our spectrum is well fit by an SN and power-law model; we do not require another component to explain the spectrum, although we explore the possibility that the afterglow contribution is lower and whether some of the resulting red excess in such a model (Methods section ‘Constraints on the afterglow contribution’) could be explained by r-process emission in the section ‘No sign of r-process enrichment’ and Methods section ‘Comparison with r-process light curve models’. Importantly, our afterglow-subtracted spectrum is similar to, although slightly fainter than, the expected flux of SN 1998bw at the distance of GRB 221009A and the phase of our observations, which suggests that the SN associated with GRB 221009A produced a similar quantity of ^56^Ni.

### A modest nickel mass indicates a typical GRB-SN

Estimates for the mass of ^56^Ni produced in SN 1998bw range from ~0.3 *M*_⊙_ to ~0.7 *M*_⊙_ depending on the models and assumptions used to fit the light curve^43–47^. Reference ^47^ considered a two-zone model where ≈0.44 *M*_⊙_ of ^56^Ni is contained in an outer high-velocity component that rapidly expands and becomes optically thin, explaining the bright peak luminosity. An additional ≈0.12 *M*_⊙_ exists in an inner dense low-velocity component that explains the linear nature of the light curve at intermediate phases of ~100–200 days.

We directly estimated the mass of ^56^Ni produced by the SN associated with GRB 221009A by integrating the afterglow-subtracted spectrum. We estimated the unobserved flux using SN 2007gr as a spectral template owing to its simultaneous optical and NIR coverage out to the same phase of our observations. We find that the wavelength coverage of our NIRSpec spectrum accounts for about 50% of the total emitted flux. At the phase of our observations, the luminosity of a nickel-powered SN is dominated by the decay of ^56^Co, the daughter isotope of ^56^Ni. Assuming a single component of the ejecta and full gamma-ray trapping, we find *M*_Ni_ ≈ 0.03 *M*_⊙_. Under a more realistic assumption of gamma-ray leakage, with a timescale of ~100 days for the ejecta to become optically thin to gamma-rays (as inferred for SN 1998bw), we find *M*_Ni_ ≈ 0.09 *M*_⊙_.

The ^56^Ni mass we infer assuming gamma-ray leakage is therefore slightly lower than the mass inferred by ref. ^47^ for the inner dense component of SN 1998bw, which is consistent with our inference that the SN associated with GRB 221009A is slightly fainter than SN 1998bw at late time. Of course, assuming a different afterglow contribution in our spectrum will affect the estimated mass. Our inferred mass is consistent with the results of ref. ^30^, who found best-fit values from modelling the early light curve of GRB 221009A in the range *M*_Ni_ = 0.05–0.25 *M*_⊙_, depending on assumptions about the host extinction, with a 99% upper limit of *M*_Ni_ < 0.36 *M*_⊙_. These values are lower than most early light curve estimates for SN 1998bw^43,45,47^. This may indicate a lower ratio of the outer-to-inner ejecta components compared with SN 1998bw, or that a two-component model is not needed to explain the SN associated with GRB 221009A. Our results, combined with the early light curve estimates, conclusively rule out the possibility that the SN was unusually bright compared with previous GRB-SNe. This is consistent with previous sample studies that do not show a correlation between the luminosities of LGRBs and their associated supernovae^24,48^. Crucially, our spectroscopic detection of the SN confirms that the marginal deviation from a typical afterglow in the early light curve claimed by ref. ^29^ and ref. ^30^ was indeed due to the SN.

### No signs of r-process enrichment

The identification of the SN associated with GRB 221009A allows us to constrain the presence of r-process material. One possibility is that the red excess in our spectrum consists of a combination of afterglow and emission from r-process elements. Reference ^15^ outlines how a collapsar with a massive transient disk can lead to r-process production. However, the observational impact of r-process material, if it is produced, is highly dependent on the degree of outward mixing. In particular, ref. ^15^ presents two possible scenarios: one (the ‘magnetohydrodynamic (MHD)’ case) in which 0.025 *M*_⊙_ of r-process elements are mixed uniformly throughout the SN ejecta with *v* < 0.15*c* and one (the ‘collapsar’ case) in which 0.25 *M*_⊙_ of r-process elements are confined to *v* < 0.015*c*. In both cases, 0.25 *M*_⊙_ of ^56^Ni is mixed in the ejecta. In the MHD case, the r-process material tracks ^56^Ni, whereas in the collapsar case, the r-process elements are embedded behind the ^56^Ni. In truth, the degree of mixing in the collapsar wind scenario is unknown, and is likely to be variable with progenitor properties and may be sufficient to mix r-process elements with the outer layers.

Although the MHD scenario has largely been ruled out by early-time observations of previous events^15,49^, few constraints exist on the collapsar wind scenario due to the lack of late-time NIR spectra of GRB supernovae. Before our NIRSpec spectrum of GRB 221009A, the latest NIR spectrum of a GRB-SN was that of SN 1998bw taken at +51 days, which we have shown is an excellent match to our spectrum (Fig. 3) after subtracting our best-fit afterglow power law. Here we consider the possibility that our best-fit power law overestimates the afterglow contribution and that our much later spectrum of the SN associated with GRB 221009A differs from the +51 day NIR spectrum of SN 1998bw owing to the presence of r-process signatures.

**Fig. 4:**
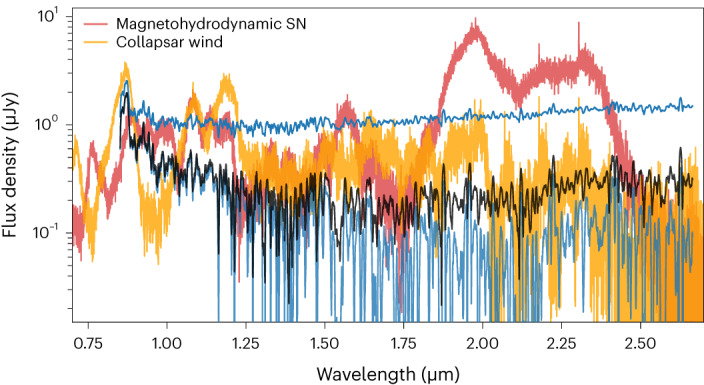
Comparison with r-process models. Comparison of our NIRSpec spectrum of GRB 221009A with r-process enriched SN models from ref. ^15^ corresponding to a phase of 95 days after explosion, which is the latest phase available. We show our original spectrum without afterglow subtraction (top blue), as well as the resulting spectra after subtracting the ALMA-XRT power law (middle black) and our best-fit afterglow model (shown in Fig. 3, bottom, light blue). Our spectrum, even after accounting for the afterglow, is clearly distinct from the predictions of an MHD SN. We also do not see evidence for spectral features in our spectrum that can be linked to the collapsar wind model and not attributed to the SN.

In Fig. 4, we compare our NIRSpec spectrum, with various assumptions about the afterglow contribution, to r-process enriched SN models from ref. ^15^ (with r-process masses up to 0.25 *M*_⊙_). We compare with models corresponding to a phase of 95 days after explosion, the latest phase available, and shift them to the distance of GRB 221009A. At this phase, the MHD SN differs considerably from an SN without r-process enrichment, producing strong emission at ≈1.8–2.4 μm that is clearly not present in our spectrum whatever the assumption on afterglow contribution. The collapsar wind model, on the other hand, largely shows SN features from non-r-process elements, although with enhanced flux near ≈2 μm compared with what is seen in normal supernovae.

Owing to the noise in our spectrum, we are unable to identify individual lines in this region of the spectrum, beyond the likely Mg i at *λ* ≈ 1.5 *μ*m. However, we compared the overall flux level and find that assuming no afterglow contribution (that is, the original unsubtracted spectrum) leads to much higher continuum flux than the collapsar wind model for *λ* > 1 μm. Furthermore, the expected strong nebular SN lines are diluted with respect to the continuum (Methods), indicating an extra continuum source is present (the afterglow of the GRB). Assuming the afterglow shape and normalization given by interpolating the contemporaneous ALMA and XRT observations, we find that, overall, the spectrum is inconsistent with the collapsar wind model, which indicates that the spectrum cannot be explained by a combination of the ALMA-XRT power law and an r-process enriched SN. Given the strong resemblance to previous supernovae across the full wavelength range when assuming our best-fit afterglow power-law shape and contribution (Fig. 3), it is unlikely that flux from r-process elements are contributing significantly to our spectrum. Our observation highlights the need for a systematic survey of nebular-phase LGRB spectra across a broad range of GRB properties, in particular, in light of the recent theoretical work that correlates these properties to the degree of r-process production^50,51^. We additionally compare our observations to the broadband colour evolution models due to r-process enrichment from ref. ^52^, which further highlights the need for spectroscopy (Methods section ‘Comparison with r-process light curve models’).

## Host galaxy properties

### A very low-metallicity, star-forming galaxy

The host galaxy of GRB 221009A is readily apparent in our JWST/NIRCam imaging shown in Fig. 1. Consistent with analysis of the optical Hubble Space Telescope (HST) images^31^, we find that GRB 221009A is located 0.24 ± 0.01 arcsec (0.66 ± 0.02 kpc assuming the cosmological parameters presented in ref. ^53^) from the centre of its host galaxy, which appears to be a near edge-on system. From our GALFIT modelling (Methods) we find that this galaxy is well described by a single Sérsic component with index *n* = 1.2 ± 0.1 and effective radius *r*_e_ = 2.15 ± 0.07 kpc. These values represent the mean and standard deviation across the four filters. The AB magnitudes in each filter corresponding to the best-fit GALFIT models are *m*_F115W_ = 21.58 ± 0.20 mag, *m*_F200W_ = 20.62 ± 0.10 mag, *m*_F277W_ = 20.88 ± 0.10 mag and *m*_F444W_ = 21.38 ± 0.05 mag (not corrected for Milky Way extinction).

**Fig. 5:**
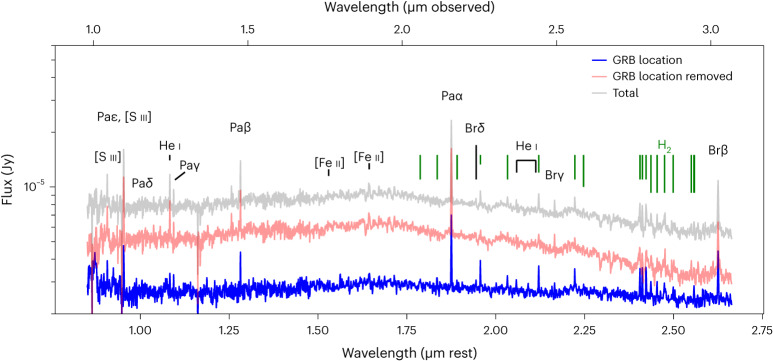
Host galaxy spectra. Spectrum of the total host galaxy including the site of the GRB (grey), the `host-only' spectrum excluding the GRB site (red; see Methods section ‘Late-time NIRSpec observations’) and a narrow aperture centred on the location of GRB 221009A (blue). We detect narrow H, H_2_, He, Fe and S emission lines from the galaxy. Importantly, we see that some narrow emission lines change in strength over this galaxy, having notably strong molecular H_2_ emission in the region of GRB 221009A. The continuum of the afterglow and SN component can be seen clearly in the blue spectrum as a deviation from the host-only spectrum.

In Fig. 5, we show the global host spectrum (that is, including flux from the entire resolved spectral trace) and the spectrum at the position of GRB 221009A (see Methods for details of the spectral extractions). We also show the ‘host-only’ spectrum, which represents an estimate of the host galaxy spectrum excluding the region of the GRB. Comparing the spectrum at the position of the GRB with the host-only spectrum clearly shows that certain lines, mostly from molecular H_2_, are much stronger at the position of GRB 221009A.

**Fig. 6:**
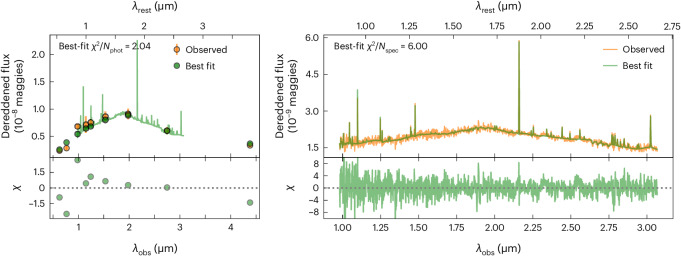
Best-fit galaxy model. Left, best-fit Prospector model photometry (green circles) and spectrum (green) compared with the observed photometry shown with 1*σ* error bars (orange circles, our NIRCam photometry and HST photometry measured by ref. ^31^). Right, best-fit Prospector spectrum compared with our NIRSpec spectrum (orange). The bottom panels present the residuals.

We measured the global host properties by fitting the global host spectrum, as well as our NIRCam photometry and HST photometry from ref. ^31^, using the stellar population modelling code Prospector^54^ (see Methods for details of the modelling procedure). The best-fit model spectrum and photometry compared with the observed data are shown in Fig. 6. We find that the host has a stellar mass of $\log (M/{{{{M}}}}_{\odot })=9.6{1}_{-0.04}^{+0.02}$ and low stellar and gas-phase metallicities of $\log ({Z}_{* }/{Z}_{\odot })=-0.8{1}_{-0.05}^{+0.04}$ and $\log ({Z}_{{{{\rm{gas}}}}}/{Z}_{\odot })=-0.9{6}_{-0.03}^{+0.09}$, respectively. This is one of the lowest metallicity environments of any LGRB, which is a class of objects that prefer low-metallicity galaxies^55–58^ and it is, to our knowledge, the lowest metallicity environment of a GRB-SN to date. This may suggest that very low metallicity is required to produce a very energetic GRB. In addition, the galaxy exhibits a recent star formation rate (SFR) of SFR_100 Myr_ = 0.17 *M*_⊙_ yr^−1^. We also find that the galaxy exhibits a visual extinction of ${A}_{\rm{V}}=0.6{7}_{-0.07}^{+0.11}$ mag. This is consistent with our extinction constraints from the early-phase JWST data (Methods) where we found a best-fit total extinction of ${A}_{\rm{V}}=4.6{3}_{-0.64}^{+0.13}$ mag, which is in good agreement, within uncertainties, with the nominal Milky Way value plus the host galaxy extinction found here. Our SFR and host extinction values are consistent with those measured from Hα and Paα detected in an early-phase X-shooter spectrum of GRB 221009A (ref. ^20^).

**Extended Data Fig. 6:**
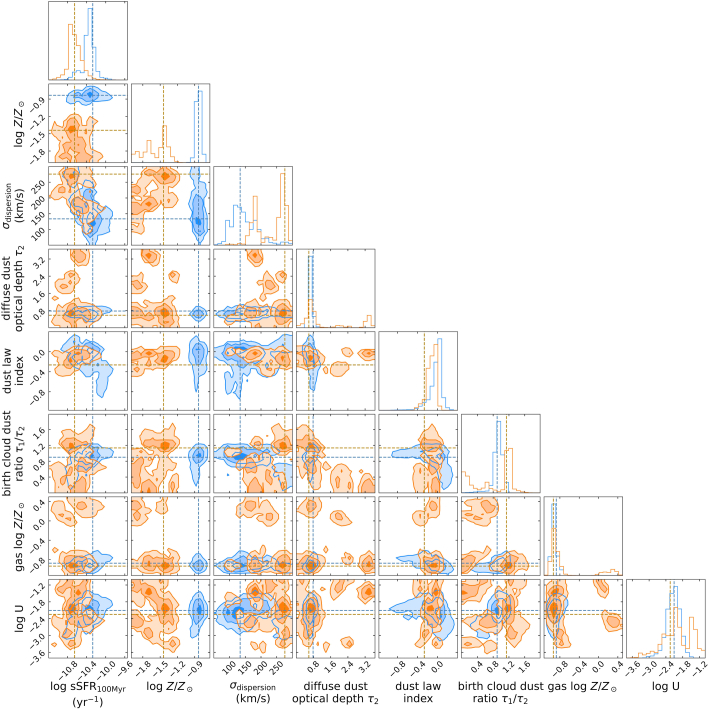
Galaxy Model Posteriors. Comparison of the Prospector model parameter posteriors for the galaxy spectra extracted at the location of GRB 221009A (orange) and the total galaxy (blue). The gas-phase metallicities are similar, but the GRB position exhibits a lower stellar metallicity.

We additionally model the spectrum at the site of the GRB, and find a similar gas-phase metallicity of $\log ({Z}_{{{{\rm{gas}}}}}/{Z}_{\odot })=-0.9{4}_{-0.06}^{+0.11}$ and a lower stellar metallicity of $\log ({Z}_{* }/{Z}_{\odot })=-1.6{6}_{-0.10}^{+0.26}$ compared with the global host galaxy (Extended Data Fig. 6), which indicates that the progenitor of GRB 221009A originated from a low-metallicity environment.

### Strong H_2_ emission at the explosion site

We observe many narrow H_2_ vibrational and rotational emission lines that appear strongest at the site of GRB 221009A, as highlighted in Fig. 5. Molecular hydrogen traces dense star-forming regions, consistent with a birth cloud of a massive stellar progenitor of a LGRB. Neglecting the afterglow itself, H_2_ can be excited by both shocks (driven by, for example, stellar winds or Herbig–Haro objects) or directly by fluorescence^59,60^. Following ref. ^61^, we compared the ratios of H_2_ lines in the range ~1.1–2.1 μm with various models of fluorescence versus collisionally excited emission from ref. ^59^ using a simple chi-squared metric (with appropriately propagated uncertainties). Owing to the strong detection of many lines (which are predicted to be absent in the case of collisional excitation), we find a better match to fluorescence models, which is consistent with the dominant excitation method in many low-metallicity, blue compact dwarf galaxies^61^. Our measured line ratios and predicted model ratios are given in Extended Data Table 2.

Only one other LGRB host, that of GRB 031203 (a relatively faint LGRB), has had a marginal detection of H_2_ emission^62^. Molecular H in absorption due to vibrational excitation has also been observed in a small number of events (see, for example, refs. ^63,64^). Statistical studies of GRB hosts have found that most lack vibrationally excited H_2_ (for example, ref. ^65^), which suggests that molecular H production is suppressed in LGRB hosts. It has been suggested that this suppression may be partially due to the low metallicities of the hosts^66^ or the ongoing star formation, leading to a strong ionizing field^67^. The low metallicity and modest SFR measured by Prospector suggests that the latter may lead to observable H_2_ emission in this event. These observations highlight the importance of the unique sensitivity and spatial resolution of the JWST when analysing the local environments of LGRB progenitors.

## Conclusions

We present the detection with the JWST of an SN associated with the highly energetic event GRB 221009A. Despite being associated with the brightest GRB ever observed, the SN produced a modest amount (≈0.09 *M*_⊙_) of radioactive ^56^Ni with no obvious signs of r-process nucleosynthesis. The host galaxy suggests a very low-metallicity progenitor system—one of the lowest metallicity environments of all known LGRBs. In addition, the exceptional sensitivity and spatial resolution of the JWST allows us to detect a series of multiple molecular H_2_ emission lines at the position of the GRB, which is an observation that has been long anticipated. A secondary site of r-process nucleosynthesis remains an open question, which can observationally be uniquely probed by late-time IR spectroscopy. Our findings motivate future JWST campaigns to examine the nebular-phase spectra of supernovae associated with LGRBs.

## Methods

### Imaging observations and photometry

We obtained imaging of GRB 221009A with the NIRCam using the F115W, F200W, F277W and F444W filters on 22 April 2023 starting at 07:08 UT. Each observation consisted of four dithered exposures with a total exposure time of 558 seconds. We downloaded the stage 3 pipeline products from the Mikulski Archive for Space Telescopes (MAST) for analysis. GRB 221009A is clearly detected, along with its host galaxy.

To measure the flux from GRB 221009A in each filter, we first modelled the host galaxy contribution using the galaxy profile fitting code GALFIT^68^. We modelled the host galaxy as a single Sérsic component. During the fit, we masked the pixels containing the light from GRB 221009A; we fitted for transient flux in a later step. The input, best-fit model and residual (best-fit model subtracted off) images are shown in Fig. 1. The residual image for the F115W filter shows no structure indicating that the galaxy light is well described by a single Sérsic component. The residual images in the three redder filters, however, exhibit remaining diffuse structure not captured by the model near the centre of the galaxy and to the northeast. Although there is no obvious evidence for such diffuse structure emanating from the position of GRB 221009A, it is plausible that GRB 221009A is co-located with a brighter region of its host galaxy that is not captured by our galaxy model. Such a determination can only be made when the transient fades.

Next, we performed point spread function (PSF) photometry on the residual images at the location of GRB 221009A. As WebbPSF only generates PSF models for use with stage 2 imaging data, we used the following custom procedure to generate drizzled PSFs for use with stage 3 data. We generated stage 2 images with model PSFs planted at the location of GRB 221009A and then ran these images through the stage 3 pipeline. We then used the drizzled PSF models for the PSF fitting of GRB 221009A. We found the following AB magnitudes in each filter: *m*_F115W_ = 25.10 ± 0.05 mag, *m*_F200W_ = 24.12 ± 0.11 mag, *m*_F277W_ = 23.77 ± 0.05 mag and *m*_F444W_ = 23.22 ± 0.08 mag (not corrected for extinction, which we assess in detail in Methods section ‘Constraints on foreground dust from early-time spectroscopy’). The uncertainties include the systematic uncertainty associated with the GALFIT modelling procedure, which we estimated by comparing PSF photometry with and without galaxy subtraction with GALFIT.

### Spectroscopic observations and data reduction

#### Late-time NIRSpec observations

We obtained spectra of GRB 221009A on 20 April 2023 with the NIRSpec^69^ onboard JWST (programme 2784; principal investigator (PI), Blanchard). Our observations began at 14:40 UT, corresponding to a rest-frame phase of 167.7 days since the Fermi GBM trigger. Spectra were taken with the S200A1 fixed slit and the medium-resolution gratings G140M/F100LP and G235M/F170LP, yielding wavelength coverage in the range ~1–3 μm. For each grating and filter set-up, we used five primary dithers and a total exposure time of 10,942 seconds. Owing to the small offset of GRB 221009A from its host galaxy^31^, target acquisition was performed using an offset star to ensure proper centring of the source in the slit.

We downloaded and inspected the pipeline products available on the MAST. A resolved trace is clearly present in the individual stage 2 exposures and final combined stage 3 products, indicating a substantial host galaxy contribution. In addition, a compact trace spanning ~2 pixels is apparent at the red end of the G140M/F100LP spectrum and the G235M/F170LP spectrum at the expected location of GRB 221009A within the slit. This trace is also at a consistent offset from the brightest part of the resolved trace representing the centre of the host galaxy, which confirms that this unresolved trace is the spectrum of GRB 221009A.

The pipeline products available on MAST were reduced using nod-subtraction, the default background subtraction method for a point source with multiple dithered exposures. Owing to the resolved nature of the overall trace, we re-reduced the data using the JWST Science Calibration Pipeline with nod-subtraction turned off to reduce the effect of subtracting source flux from itself. We then extract the one-dimensional spectrum of GRB 221009A from our re-reduced stage 3 combined and rectified two-dimensional (2D) spectra for further analysis. The final 2D spectra are shown in Extended Data Figs. 1 and 2 (see Fig. 1 for the slit orientation).

We used the following extraction procedure to isolate the flux associated with GRB 221009A from the light of its host galaxy. We modelled the spatial profile of the overall trace as a two-component Gaussian with centres fixed at the position of GRB 221009A and the centre of the host galaxy. We fitted the total spatial profile, summed over all wavelengths, to determine the best-fit Gaussian width of each component. We then fitted this model, with widths fixed at these values, to the spatial profile at each wavelength. We also fitted for a linear background component determined from background regions located on both sides of the trace. The sum of the flux in each fitted Gaussian component thus represents the flux from GRB 221009A and its host galaxy as a function of wavelength. The resulting spectra of GRB 221009A in the G140M and G235M gratings are shown in Extended Data Figs. 1 and 2, respectively. We also show corresponding error spectra calculated from the 2D pipeline-generated error arrays for each grating. The fit to the host galaxy Gaussian component yields a ‘host-only’ spectrum.

We note that the background exhibits evidence for PSF artefacts that are potentially from a nearby bright star (the pseudoperiodic signal at pixel rows ~18–24 in the 2D frames; Extended Data Figs. 1 and 2), which is the most likely explanation given the crowded nature of the field. Owing to the difficulty of accurately modelling this component of the background, our background regions exclude those containing such artefacts. Our fitted background therefore represents the smooth underlying sky background. This may mean that the background at the location of GRB 221009A and its host galaxy is underestimated. However, we extract regions of the background containing the suspected PSF artefacts from a nearby star and find no evidence that these features are present in our extracted spectrum of GRB 221009A. In addition, the flux from these features decreases towards the spatial location of the GRB spectral trace.

#### Combined G140M + G235M spectrum compared with photometry

As the photometry was obtained only two days after our NIRSpec spectra, we used the photometry to check the flux calibration of the spectra. We find that the fluxes in the four NIRCam filters are an excellent match to the flux calibration of the G140M and G235M spectra. In addition, the two spectra agree in the wavelength region where the gratings overlap. In Fig. 2, we show the fluxes in each filter compared with the combined G140M + G235M spectrum.

#### Host galaxy spectral extractions

As seen in Extended Data Figs. 1 and 2, the host galaxy is resolved in our JWST/NIRSpec observations, extending across approximately ten rows in our 2D spectra and with numerous narrow emission lines. To study the global host properties, we extracted the entire trace including the position of the GRB. We note that there is significant variation of the strength of some emission lines across the spatial extent of the galaxy, where several lines are stronger at the position of GRB 221009A. To identify these lines and assess any potential variation in the galaxy properties at the position of the GRB, we extracted a narrow aperture centred on the position of GRB 221009A. This differs from the Gaussian decomposition procedure described in Methods section ‘Late-time NIRSpec observations’ used to isolate the GRB spectrum, as here we are not modelling and subtracting the underlying host spectrum; the goal here is to measure the host properties at the position of the GRB.

#### Archival NIRSpec/MIRI observations

We obtained archival spectroscopic observations of GRB 221009A from JWST, observed with NIRSpec and the MIRI on 22 Oct 2022 (programme 2782; PI, Levan and originally presented in ref. ^31^). These observations correspond to 13.16 and 13.2 days post burst, respectively.

At this epoch, the NIRSpec observations were taken in the low-resolution PRISM mode, with spectral coverage from ~0.5 μm to ~5.5 μm. The pipeline products from MAST reveal a clear, high signal-to-noise ratio trace in the 2D spectrum. The stage 3, reduced spectrum is consistent with that published in ref. ^31^, and we thus use it for analysis in this work without additional reductions.

The MIRI spectrum was taken in the low-resolution spectroscopy mode with the P750L disperser. The automatic reduction of the MIRI spectrum failed, which was likely to be due to improper selection of the afterglow trace. We used the official MIRI reduction pipeline to manually extract the spectrum from the stage 2 products, carefully selecting the correct trace and appropriate background from the nodded 2D image. At this epoch, the afterglow trace is clearly identified in the 2D spectrum and easily isolated using a simple boxcar extraction. We note that MIRI is uncalibrated below *λ* ≲ 4.5 μm at the time of analysis, and we therefore removed data below this wavelength of the spectrum from analysis. The MIRI observations are qualitatively consistent with those of ref. ^31^ and are well matched to their near-simultaneous photometric observation in F560W.

### ALMA observations

Following the seven epochs of ALMA observations of GRB 221009A through programme 2022.1.01433.T (PI, Laskar), we obtained two additional epochs with the same programme on 01 March 2023 at a mean time of 15:41 UT and on 11 April 2023 at a mean time of 07:55 UT, corresponding to 143.6514 and 183.7838 days in the observer frame, respectively (≈124.80 and ≈159.67 days in the rest frame). Both observations utilized two 4 GHz wide base-bands centred at 91.5 and 103.5 GHz, respectively with J1924 − 2914 as bandpass calibrator and J1914 + 1636 as complex gain calibrator. The millimetre-band afterglow previously reported in ref. ^25^ was clearly detected in the pipeline-processed science-ready data products in the first of the two epochs and more weakly (≈4.7*σ*) detected in the second epoch. We performed photometry using imfit in the Common Astronomy Software Applications^70^ and find a best-fit flux density in the two epochs of (163 ± 22) μJy and (104 ± 23) μJy (including a 5% systematic flux calibration uncertainty) at a mean frequency of 97.5 GHz, along with a position of RA = 19 h 13 m 03.50 s and dec.= 19^∘^46′24.3′′ with an uncertainty of 0.1^*″*^ in each coordinate (consistent across both epochs). Together with the last ALMA 97.5 GHz measurement reported in ref. ^25^, the temporal decline rate of the millimetre-band afterglow at ~99–188 days after the burst (observer frame) is *α*_mm_ = −1.54 ± 0.08, which implies an extrapolated millimetre-band flux density of (99 ± 23) μJy at the time of the NIRSpec observations (194 days, observer frame).

### Swift/XRT observations

We downloaded the count-rate light curve of the X-ray afterglow of GRB 221009A from the Swift/XRT website^71^. Using the spectral parameters presented in ref. ^25^ (Milky Way (MW) absorption of *N*_H,MW_ = 5.36 × 10^21^ cm^−2^, intrinsic absorption of *N*_H,int_ = 1.35 × 10^22^ cm^−2^ and photon index of *Γ*_X_ = 1.8566), we converted the observed count rate to a flux density (*F*_X_) at 1 keV and obtained *F*_X_ = (9.3 ± 3.5) × 10^−3^ μJy at $19{6}_{-9.1}^{+5.5}$ days (observer frame; corresponding to $170.{4}_{-7.9}^{+4.9}$ days, rest frame) after the burst. Comparing this with our ALMA observations, we find that the spectral index between the ALMA and XRT observations at the time of the NIRSpec observations (≈194 days, observer frame) is *β*_ALMA-XRT_ = 0.63 ± 0.03. We refer to this number elsewhere in the text as the ALMA-XRT power law, anchored to the inferred ALMA flux density of ≈0.1 mJy at the time of the NIRSpec observations.

### Constraints on foreground dust from early-time spectroscopy

Given the location of GRB 221009A in the Galactic plane (*b* ≈ 4^∘^), we expected substantial extinction due to interstellar dust in the MW. Reference ^36^ estimates the MW extinction contribution to be *A*_V_ = 4.10 ± 0.06, assuming the standard extinction factor *R*_V_ = 3.1. As noted in ref. ^72^, these dust maps can be unreliable for low galactic latitudes^73^. Furthermore, this measurement neglects host contribution; however, given the relatively low redshift, we do not expect to easily distinguish between dust arising from either the MW or host galaxy (see, for example, the results of ref. ^20^). For simplicity, we neglect redshift dependence of dust.

Given the significant uncertainties expected, we opted to use the first epoch of NIRSpec/MIRI data to determine the appropriate extinction correction. We assumed that the spectrum is dominated by some unknown combination of an afterglow (power-law model) and a thermal, SN-like component. Unless r-process material is mixed significantly within the ejecta, we do not expect a red thermal component at early times. As such, we assumed that the event is dominated by a power-law afterglow at *λ*_obs_ ≳ 3 μm; below this wavelength, it is reasonable that a SN 1998bw-like event could contribute significant flux. We explore the systematic uncertainties associated with the extinction laws and assumptions on the SN contribution.

Few extinction laws are calibrated across the full wavelength range covered by the NIRSpec/MIRI observations; a new extinction law describing *A*(*λ*)/*A*(V) as a function of *R*(V) in the range ~0.1–30 μm has been recently presented^38^. We contrast this solution with the commonly used extinction law described in ref. ^37^ to quantify systematic uncertainty from assumptions of dust laws. Given a prescribed dust law, we simultaneously fitted the observed day 13.2 (observer frame) spectrum to a power law (*F*_*ν*_ ∝ *ν*^−*β*^) and extinction parameters *A*_V_ and *R*_V_ using a Markov chain Monte Carlo sampler implemented in emcee^74^. Our models have four free parameters: the overall power-law normalization (‘norm’), the power-law index *β*, the dust *A*_V_ and *R*_V_ values, and a white noise scatter term. The scatter quantifies the uncertainty in JWST flux estimates as a fraction of the flux. We assumed a wide uniform prior for all parameters except normalization, in which we assumed a log–uniform prior.

We first fitted using the dust law presented in ref. ^38^. Fitting all observed wavelengths *λ*_obs_ < 8 μm, we find *β* = 0.39 ± 0.01, *A*_V_ = 4.37 ± 0.05 and ${R}_{\rm{V}}=3.0{7}_{-0.05}^{+0.04}$. At *λ* < 2 μm, we find that the residuals are consistent with 0, suggesting no contribution from an additional thermal component. We next excluded wavelengths <2 μm in the fitting process to test the possibility of contamination from either a SN-like or r-process thermal event. We find that when excluding these wavelengths, the afterglow model overestimates the blue flux.

Next, we fitted using the extinction law described in ref. ^37^ (that is, following the original analysis of ref. ^31^). Again, we emphasize that this extinction law is not calibrated for IR observations and simply extrapolates at these wavelengths. We simultaneously fitted the observed spectrum (*λ*_obs_ < 8 μm) to a power law and extinction model. We find *β* = 0.41 ± 0.01, ${A}_{\rm{V}}=4.6{3}_{-0.64}^{+0.13}$ and ${R}_{\rm{V}}=4.2{4}_{-0.64}^{+0.74}$. This is significantly different (>3*σ*) from the results presented in ref. ^31^ when only accounting for statistical uncertainties, which we attribute to a tight prior (versus our flat prior) set by those authors.

We report the results of our fits in Extended Data Table 1 and show these data, models and associated residuals in Extended Data Fig. 3. The residuals of both dust models show significant structure throughout the spectrum. We specifically compared the residuals to a spectrum of SN 1998bw taken 12 days post burst and scaled to the redshift of GRB 221009A. We note that the statistical uncertainties and systematic difference between these two dust extinction models mean that we are unable to make a conclusive statement on the SN emission from the early-time JWST spectrum. This is a different conclusion from that of ref. ^31^, who, given their small statistical uncertainties, rule out SN 1998bw-like thermal emission at early times.

### Constraints on the afterglow contribution

#### Initial comparisons with previous supernovae

In Extended Data Fig. 5, we show our extinction-corrected spectrum (using the law in ref. ^37^ and best-fit parameters listed in Extended Data Table 1) compared with spectra of SN 1998bw^40^, the canonical SN Ic-BL associated with a GRB and SN 2013ge^42^, one of the few supernovae Ic with high S/N late-time NIR spectra, taken at +51 and +118 days after peak, respectively. To achieve complete overlap with the blue end of our spectrum, we combined the +51 day NIR spectrum of SN 1998bw with an optical spectrum taken at +73 days. We scaled the spectra of SN 1998bw and SN 2013ge to the distance of GRB 221009A and used their light curves^41,42^ to normalize to their brightnesses at the phase of our GRB 221009A spectrum. Our spectrum of GRB 221009A is brighter than the comparison supernovae would be and relatively featureless with a different overall spectral shape, which is consistent with significant contamination from the afterglow. Our spectrum exhibits flux increasing at *λ* ≳ 1.5 μm, whereas the comparison supernovae exhibit declining flux.

The emission features shown in Extended Data Fig. 4 exhibit similar, although slightly narrower, widths than the corresponding features in SN 1998bw, SN 2013ge and SN 2014ad. Owing to the lack of a late-time light curve for SN 2014ad, we scaled its spectrum to roughly match SN 1998bw for comparison purposes. In addition, the lines in our JWST spectrum are diluted in strength and exhibit a different flux ratio. This, combined with the rising flux to the red, means that there is no simple luminosity scaling that will bring our spectrum of GRB 221009A into agreement with the comparison spectra. These observations are consistent with afterglow contamination. Furthermore, the lack of many strong SN features (for example, the strong P-Cygni features near ≈1 μm, ≈1.5 μm and ≈2 μm commonly seen in supernovae^39^) other than the two identified (Ca ii NIR triplet and O i at ≈0.86 μm and ≈0.92 μm, respectively) indicates that the SN associated with GRB 221009A is not substantially brighter than SN 1998bw and SN 2013ge.

#### Constraints from contemporaneous ALMA and Swift/XRT observations

Determining the afterglow contribution is critical for constraining the presence of SN emission and a possible contribution from r-process material. First, we considered the power law formed by the ALMA and XRT observations that we obtained around the same phase as our JWST observations. We analysed the residual spectrum by subtracting off the ALMA-XRT power law from our spectrum of GRB 221009A which we show, compared with SN 1998bw and SN 2013ge, in Extended Data Fig. 5. Although the resulting spectrum matches more closely the shape of the supernovae compared to the unsubtracted spectrum, in particular at the blue end, the shape at *λ* ≳ 1.5 μm still exhibits rising flux substantially different from the supernovae. Given the lack of strong emission features in this region, the most likely explanation is that the ALMA-XRT power-law model does not adequately capture the afterglow contribution. In section ‘No signs of r-process enrichment’ and Methods section ‘Comparison with r-process light curve models’, we consider whether this red excess could be due to emission from r-process material.

#### Varying the afterglow contribution

Next we considered the best-fit power law from fitting our spectrum at *λ* ≳ 1.5 μm (shown in Fig. 3) and analysed how the implied SN component changes with different afterglow normalizations. We scaled the best-fit power law by factors of 0.3, 0.6, 0.9 and 1.0 to generate four potential afterglow models, subtracted them from the spectrum and compared the resulting residual spectra with SN 1998bw and SN 2013ge. In Supplementary Fig. 1, we show the residual spectra and afterglow models for the four scalings. When scaled by 0.3 and 0.6, the residual spectra still exhibit flux rising to the red, as in the unsubtracted spectrum, which indicates that these models are not likely to account for all of the afterglow flux.

In addition, there is a mismatch between the flux ratios of the expected emission lines. In other words, the detection of the Ca ii NIR triplet at the strength we see, would imply the detection of other lines at strengths that are not observed. Of course, this reasoning relies on the assumption that the SN associated with GRB 221009A should appear similar to previous supernovae Ic/Ic-BL. Indeed it is possible that this SN may not show the same features as previous events and potentially an additional component from r-process emission which we assess in section ‘No signs of r-process enrichment’ and Methods section ‘Comparison with r-process light curve models’. However, the lack of strong lines in this region indicates that the SN associated with GRB 221009A is likely to be fainter than these models suggest and the afterglow is correspondingly brighter (as found when performing a joint SN + afterglow fit; see section ‘Isolating the SN signal’), such that most emission lines are diluted with respect to the continuum and are not detectable.

If instead the best-fit power law is scaled by 0.9, the residual spectrum appears consistent with the comparison spectra and is a close match to the overall flux level of SN 1998bw. Note that this is similar to the best-fit scaling (0.94) when performing the joint SN + afterglow fit as described in ‘Isolating the SN signal’ and shown in Fig. 3. Larger afterglow contributions (for example, scaling by 1.0) yield an overall steeper slope, inconsistent with the comparison objects.

### Comparison with r-process light curve models

We also considered the r-process enriched SN light curve models in ref. ^52^. In Supplementary Fig. 2, we show the J–H and J–K colour evolution of these models, for an SN Ic-BL with a typical simulated ejecta mass of 3.96 *M*_⊙_, a ^56^Ni mass of 0.33 *M*_⊙_, an r-process material mass of 0.03 *M*_⊙_ and various levels of r-process mixing, from no mixing to nearly fully mixed, compared with the colours of the SN component of GRB 221009A under different afterglow assumptions. We calculated J–H and J–K colours by convolving the filter bandpasses with our NIRSpec spectrum after subtracting the afterglow models. We show the resulting colours for the afterglow models considered in section ‘Constraints on the afterglow contribution’ (the ALMA-XRT power law and the best-fit power law from fitting the red end of our spectrum with various normalizations; Extended Data Fig. 5 and Supplementary Fig. 1).

The J–K colours of the afterglow-subtracted spectra match the r-process enriched models when scaling the best-fit power law by ≲0.9. Decreasing the afterglow contribution leads to more residual red light, leading to redder colours. When scaling by ≲0.6, including the ALMA-XRT model, the J–K colours, if reddened due to r-process material, would imply significant mixing. In this case, strong broad emission lines from r-process elements would be expected, as seen in the MHD model in Fig. 4 but not in our data. In addition, for a given afterglow contribution, the J–H colours imply a different degree of r-process mixing than the J–K colours, which suggests that the reddening source is not due to r-process emission.

In Supplementary Fig. 2, we also show the colours of SN 1998bw and SN 2013ge calculated from their late-time NIR spectra. SN 1998bw is notably blue—bluer even than the models without r-process—which suggests that these models do not fully capture the range of possible spectral energy distributions of typical GRB supernovae. SN 2013ge is notably red, which is consistent with the r-process enriched models for a mixing fraction of ~10%. This event, however, exhibits a clear example of carbon monoxide emission increasing the flux in the K-band. These comparisons highlight that, without spectra, other sources of reddening are difficult to disentangle from that due to r-process material. Similar conclusions have been drawn from studies of large samples of supernovae Ic-BL light curves^49^. We note that the spectrum of GRB 221009A after subtracting the best-fit power law scaled by 0.9, which yields a good visual match to SN 1998bw and SN 2013ge (Supplementary Fig. 1), exhibits a J–K colour that is ~0.1 mag redder than the no r-process model. However, as can be seen in Supplementary Fig. 1, there is an upturn in the spectrum in the K-band at the expected location of the first overtone carbon monoxide emission, similar to that seen in SN 2013ge.

### Host galaxy modelling

We used Prospector^54^, a Bayesian galaxy spectral energy distribution (SED) fitting code to simultaneously fit the global host galaxy photometry and spectroscopy. Additionally, we fitted the spectrum extracted at the position of the GRB to compare the global host properties and those at the GRB position. We adopted the MIST isochrones^75^ and the C3K stellar spectral libraries in the Flexible Stellar Population Synthesis^76,77^ framework. The stellar population is described by redshift, stellar mass, velocity dispersion, stellar metallicity and a step function non-parametric star formation history with 14 time bins^78^. The nebular emission was parametrized by gas-phase metallicity and ionization parameter using the CLOUDY grid in ref. ^79^. We simultaneously fitted simple Gaussians to lines that are not included in our emission line model that assumes that all the emission is powered by the stars, namely, the He i, [Fe ii] and H_2_ emission lines, with the same kinematics but free amplitudes as our CLOUDY grid. We assumed a flexible two-component dust attenuation model that accounted for birth cloud and diffuse dust separately^80^. Variation in the shape of the attenuation curve was enabled using a power-law modification to a Calzetti curve^81^. We also incorporated the contribution of dust emission to the infrared photometry using a three-parameter model^82^. To fit the spectroscopy and photometry together, we marginalized over the shape of the observed spectrum (thereby avoiding any wavelength-dependent flux calibration issues) with a polynomial; in this manner, the normalization and shape of the SED was entirely determined by the photometry, or not constrained at all for the GRB position, where there is no photometry. We also included a jitter parameter that inflated the spectroscopy uncertainties to account for imperfect JWST flux calibration and slit losses, and found typical values of 1.5–2, which are consistent with other early JWST spectroscopic analyses^83–85^. Finally, we used a pixel outlier model to downweight pixels that were not consistent with our model^86^, which were typically identified at a 1–2% level. In summary, the SED model for the host galaxy fit has 28 free parameters, and the fit to the spectrum at the GRB position has 24 free parameters.

### Supplementary information

Supplementary Information Supplementary Figs. 1 and 2.

## Figures and Tables

**Extended Data Table 1:**
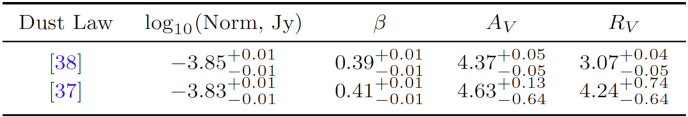
Extinction Fitting Results

Best-fit extinction parameters and 1*σ* uncertainties from fitting the +13.2 day (observer frame) NIRSpec/MIRI data.

**Extended Data Table 2:**
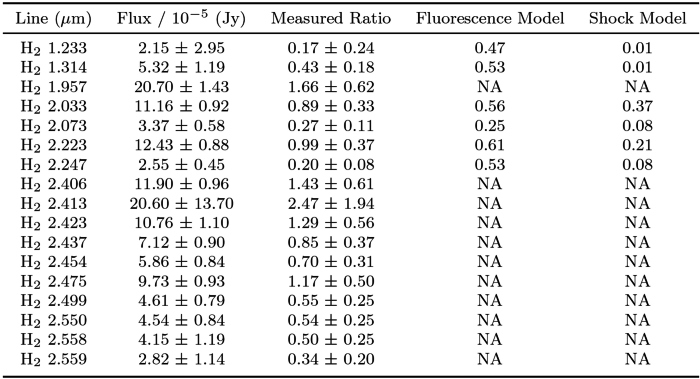
H_2_ Emission Line Measurements

H_2_ emission line fluxes and ratios with respect to H_2_ 2.122 *μ*m (with flux (8.34 ± 3.50) × 10^−5^ Jy). Predicted ratios from the shock and fluorescence models are also given.

## Data Availability

The JWST data analysed in this work associated with programmes 2784 and 2782 are publicly available on the MAST archive.

## Code availability

The software tools used in this work (JWST Science Calibration Pipeline, GALFIT, WebbPSF, Prospector and Common Astronomy Software Applications) are publicly available.
