# Supplementary material for: JWST detection of a supernova associated with GRB 221009A without an r-process signature
Source: Nat Astron. 2024 Apr 12;8(6):774–85. doi: 10.1038/s41550-024-02237-4 (PMC11189819; doi:10.1038/s41550-024-02237-4)
Supplement: Supplementary file 1 — Supplementary Figs. 1 and 2. [file 41550_2024_2237_MOESM1_ESM.pdf]

nature astronomy

Article https://doi.org/10.1038/s41550-024-02237-4

JWST detection of a supernova associated with GRB 221009A without an r-process signature

In the format provided by the authors and unedited

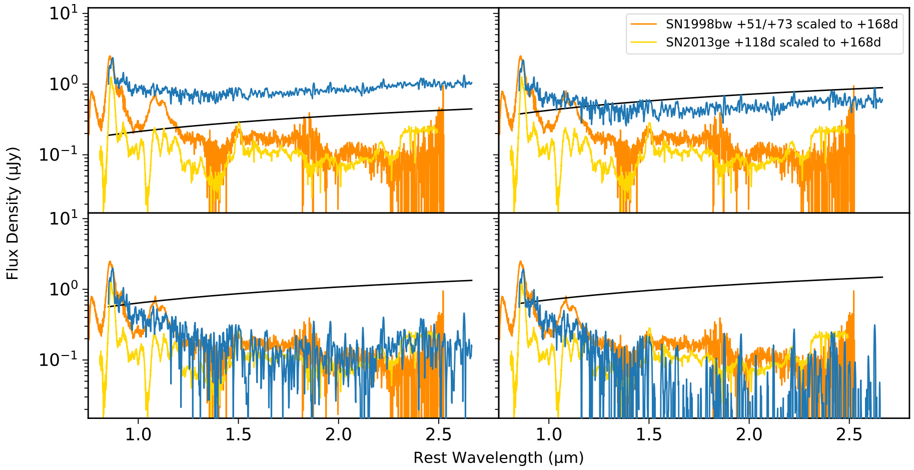

Supplementary Figure 1: Varying the Afterglow Contribution. Our extinction-corrected NIRSpec spectrum of GRB 221009A (blue; smoothed) with various afterglow models subtracted compared to ground-based late-time NIR spectra of SN 1998bw (orange) and SN 2013ge (gold) scaled to the distance of GRB 221009A and the same phase of our observations. In each panel, we show our spectrum of GRB 221009A after subtracting our best-fit model for the afterglow (black curve) described by the power law $F_\nu \propto \nu^{-0.76\pm0.07}$ and scaled by factors of 0.3 (Top left), 0.6 (Top right), 0.9 (Bottom left), and 1.0 (Bottom right).

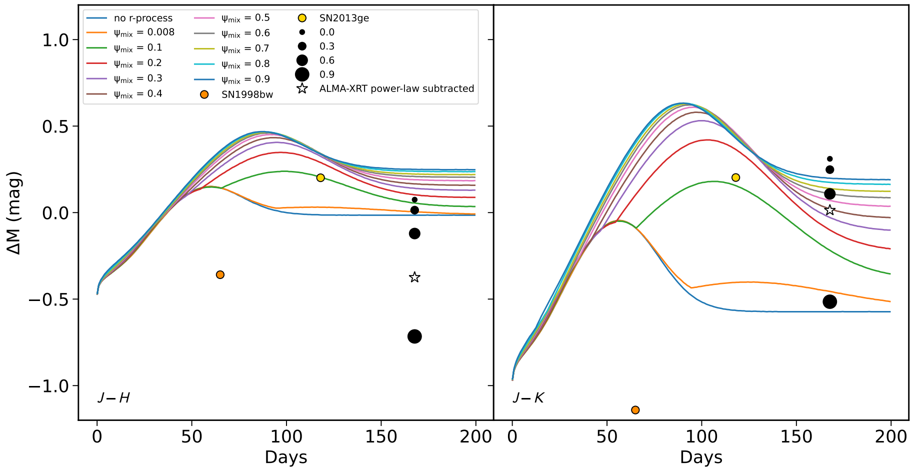

Supplementary Figure 2: Comparisons with Additional r-Process Models. Comparison of the $J-H$ (left) and $J-K$ (right) colors of GRB 221009A, calculated from our +168 day NIRSpec spectrum *after* subtracting various afterglow models (star and black points), compared to the predicted color evolution of r-process enriched SNe from the models of [52] (lines, with ψ representing the degree of mixing). We show the colors of the SN component of our spectrum of GRB 221009A assuming the ALMA-XRT power law for the afterglow (star; see Extended Data Figure 5) as well as our best-fit afterglow model from fitting the red end of our spectrum scaled by factors of 0.0 (i.e. no subtraction), 0.3, 0.6, and 0.9 (black points; see Supplementary Figure 1). We also show the colors of SN 1998bw (orange point) and SN 2013ge (yellow point) calculated from their spectra.
